# Objective and subjective diagnostic parameters in the fellow eye of unilateral keratoconus

**DOI:** 10.1186/s12886-017-0584-2

**Published:** 2017-10-06

**Authors:** Eman A. Awad, Waleed A. Abou Samra, Magda A. Torky, Amr M. El-Kannishy

**Affiliations:** Ophthalmology Center, Faculty of Medicine, Mansoura University, 24 Al-Gomhoria street, Mansoura, Egypt

**Keywords:** Pentacam, Forme fruste, Ambrósio relational thickness, Contrast sensitivity and glare

## Abstract

**Background:**

Keratoconus (KC) is usually a bilateral corneal ectatic disease. For significant asymmetric presentation (so called unilateral KC), the fellow eye has the mildest and earliest form of the disease, which is typically called forme fruste keratoconus. The aim of this study was to evaluate the sensitivity and specificity of parameters derived from a Scheimpflug imaging system (Pentacam) as well as the changes in the quality of mesopic vision in the apparently normal fellow eye (forme fruste) to detect the earliest and most sensitive parameters.

**Methods:**

Patients with clinical keratoconus in one eye and forme fruste keratoconus in the fellow eye were compared to subjects with normal eyes. The patients were examined using a rotating Scheimpflug imaging system (Pentacam).The following parameters were evaluated: keratometry, minimum corneal thickness, pachymetry progression index (PPI), Ambrósio relational thickness (ART), posterior elevation, back difference elevation (BDE) and multimetric D index(D index). Receiver operating characteristic (ROC) curves were analyzed by evaluating the area under the curve (AUC) to detect the sensitivity and specificity of each parameter. Mesopic vision evaluations were performed by contrast sensitivity and glare tests for each group.

**Results:**

A total of 48 patients with clinical keratoconus in one eye and forme fruste keratoconus in the fellow eye and 72normal subjects were evaluated. In the clinical keratoconus eyes, the mean K, back difference elevation (BDE), pachymetric progression index maximum(PPI max), and multimetric D were significantly higher compared to the normal subjects, whereas the corneal pachymetry and Ambrósio relational thickness maximum (ART max) were significantly lower. In the forme fruste eyes, the ROC analysis showed that the AUC values of the mean K, thinnest pachymetry, ARTmax, BDE, D index, and PPI max were 0.82, 0.61, 0.88, 0. 67, and 0.64, respectively. The contrast sensitivity and glare tests were significantly affected in the forme fruste cases.

**Conclusion:**

In forme fruste keratoconus eyes, the ART max is considered a highly sensitive objective parameter. Contrast sensitivity and glare is an important subjective test, which is affected in forme fruste patients.

## Background

Keratoconus is a progressive corneal ectatic disorder that may have variable presentations especially in earlier stages. The disease is usually bilateral but with a high prevalence of inter-eye asymmetry. In certain cases of significant asymmetric presentation (so called unilateral keratoconus), the fellow eye may show the earliest and mildest form of the disease, corresponding to the definition of forme fruste keratoconus. There have been some reports that patients with unilateral keratoconus will eventually develop keratoconus in the fellow eye due to genetic causes [1, 2].Therefore, early detection of forme fruste keratoconus is very important.

Keratoconus should be diagnosed in the early stages, as studies have suggested that UV-A mediated cross-linking may stop the progression of the disease and preserve visual function [3, 4]. Moreover, discrimination of early keratoconus among refractive candidates may reduce the risk of post-Lasik ectasia, which is considered one of the most feared postoperative complications of refractive surgery [5].

The Pentacam imaging system provides several keratometric, pachymetric, and elevation parameters as well as the Belin Ambrósio deviation index with different sensitivity and specificity [6, 7].

All parameters should be evaluated to determine the most sensitive and specific parameters of early keratoconus.

The aim of this study was to evaluate and compare different Pentacam parameters. Moreover, the mesopic vision in patients with keratoconus in one eye and forme fruste keratoconus in the fellow eye was assessed to facilitate the diagnosis of this condition.

## Methods

This cross-sectional study was carried out on patients presenting keratoconus in one eye and forme fruste keratoconus in the fellow eye; the patients attended the outpatient clinic of Mansoura ophthalmic center between January 2014 and February 2015. This study was approved by the institutional research board of the Mansoura faculty of medicine and was performed in accordance with the ethical standards of the Declaration of Helsinki. All patients included in the study provided informed consent.

Ocular examinations were performed on all eyes included in the study. Clinical Keratoconus was diagnosed if all the following criteria were found:1) asymmetric bow tie with skewed radial axis (SRAX), inferior or central steepening on the topography map, 2) mean keratometry (K) > 47diopters or an inferior–superior (I-S) value >1.4diopters according to the Rabinowitz and McDonnell criteria [8], and 3) at least one clinical symptom(i.e., stromal thinning, conical protrusion of corneal apex, Fleischer ring, Vogt striae or anterior stromal scar). Forme fruste keratoconus was diagnosed if all the following criteria were found: 1) normal topography, 2) mean K < 47D and I-S ≤ 1.4, 3) normal slit lamp, and 4) keratoconus in the fellow eye.

The eyes with an evident keratoconus diagnosis were included in (Group A) and the fellow eyes were included in (Group B).Control cases(Group C) were selected from the normal database of candidates for refractive surgery with the following:1)normal topography, 2) negative rotating Scheimpflug device indices and negative topographic keratoconus classification, 3) normal slit lamp examination, and 4) no history of eye disease(only one eye of each normal candidate was included in the study depending on a computer-based randomizing program).

The exclusion criteria included previous ocular surgery or trauma, significant corneal scarring or associated ocular pathology.

All eyes were examined by rotating Scheimpflug corneal tomography (Pentacam, Oculus Optikgerate GmbH).No patients in the study wore contact lenses. During the Scheimpflug corneal tomography examination, the patient was properly positioned on the chin rest and forehead strap. The patient was asked to blink several times then open both eyes and stare at a fixed target. After obtaining proper alignment, the automatic release mode started the scan; a total of25 single Scheimpflug images were captured within 2 s for each eye. Three consecutive scans were taken of each eye by the same examiner. The average of the results of three acceptable measurements was included in the study. Each eye was required to have a corneal map with at least 9 mm of corneal coverage and no extrapolated data.

The Pentacam maps were analyzed. The following anterior and posterior corneal surface parameters were evaluated by the Scheimpflug system: corneal dioptric power at the flattest meridian in the 3 mm-central zone(K1), corneal dioptric power in the steepest meridian in the 3 mm-central zone (K2) and mean corneal power in the 3 mm-zone (mean K).

The pachymetric map was analyzed, including the central corneal thickness (CCT) at the apex of the geometric center and corneal thickness at the thinnest point (CTmin).The average progression index (PPI avg) was calculated as the progression value at the different rings referenced to the mean curve. The minimum (PPI min) and maximum (PPI max) progression indices were recorded. The Ambrósior elational thickness (ART) was calculated as follows: ARTavg = CTmin/PPIavg, ARTmin = CTmin/PPImin, ARTmax = CTmin/PPImax [9].

The posterior corneal elevation maps were evaluated; a reference best fit sphere was calculated at a fixed optical zone of 8 mm, and the posterior corneal elevation values relative to this reference were recorded. The back difference elevation and multimetric D index values were extrapolated from the difference map of the Belin/Ambrósio-enhanced ectasia display of the Pentacam system.

### Contrast and glare sensitivity test

The contract and glare tests were carried out with a Mesotest II (Oculus Optikgeräte GmbH, Wetzlar, Germany), which consists of Landolt rings of different contrast levels presented in front of a low-brightness background. There are 4 contrast levels, 1:23, 1:5, 1:2.7, and 1:2, which represent the ratio between the light intensity of the optotypes and the background. There were 8 tests, 4 without glare and 4 with glare. Test 1, with a contrast level of 1:23, is the most easily recognized. For statistical purposes, each level of the contrast or glare test was given a score ranging from 20% at the 1:23 level to 100% at the 1:2 level.

### Statistical analysis

Data entry and statistical analyses were performed using SPSS version 20.0 (SPSS Inc., Chicago, IL, USA). The normality of the data was first tested by a one-sample Kolmogorov-Smirnov test. The parametric data were expressed as mean ± standard deviation. In addition, a one-way ANOVA test was used to compare the means of continuous parametric variables for three different groups; then, post hoc tests were performed to compare each set of two groups. A receiver operator characteristic (ROC) curve was used to illustrate and compare the diagnostic accuracy of each measured parameter in the keratoconus eyes; the selection of cut off values was based on the best sensitivity and specificity accepted by the researchers. A *P*-value of <0.05 was considered statistically significant.

## Results

A total of 48 patients attended the Mansoura ophthalmic center and were diagnosed with clinical keratoconus in one eye (GroupA) and forme fruste in the fellow eye (Group B).The control cases (Group C) included 72 eyes of normal refractive candidates.

The mean age of the keratoconus and control groups was 30.6 ± 9.2 years (range 14 to 44 years) and 26.6 ± 6.2 years (range 18 to 38 years), respectively. There was no significant difference in age between the two groups (*P* = 0.209).

Table 1 shows the mean keratometric values and the pachymetric and posterior elevation parameters for all groups.

**Table 1:** Comparison of Scheimplug parameters between the keratoconus, forme fruste, and control group

Parametrer	KCN	FFKC (fellow eye)	Control	KCN vs Cont	FFKC vs Cont	KCN vs FFKC
Ks	46.75 ± 2.2	42.37 ± 1.2	42.3 ± 1.3	<0.001	0.3	<0.001
Kf	43.17 ± 2.02	41.94 ± 1.3	41.9 ± .8	<0.001	0.5	0.002
Km	44.68 ± 1.7	43.01 ± 1.15	42.05 ± 1.17	<0.001	0.04	<0.001
CCT	494 ± 37.2	519 ± 31	520 ± 28.24	<0.001	0.04	0.002
CT min	464.17 ± 10	514.57 ± 5.8	516 ± 4.4	<0.001	0.1	<0.001
CCT-CT min	32.7 ± 4.3	5.6 ± 3.34	4.7 ± 2.14	<0.001	0.869	<0.001
PE	25.6 ± 16.4	6.3 ± 3.2	5.6 ± 1.5	<0.001	0.02	<0.001
BDE	22.9 ± 11.7	6.39 ± 2.4	3.35 ± 1.6	<0.001	0.04	<0.001
PPI avg	1.8 ± 0.56	1.07 ± 0.09	0.11 ± .018	<0.001	0.3	<0.001
PPI min	1.24 ± 0.39	0.65 ± 0.0.18	0.7 ± 0.13	<0.001	0.1	<0.001
PPI max	25.64 ± 16.4	1.42 ± 0.2	6.3 ± 3.2	<0.001	<0.001	<0.001
ART avg	280 ± 102.2	480 ± 42.4	478.8 ± 49.5	<0.001	0.6	<0.001
ARTmin	481.4 ± 264	832.01 ± 199.4	927 ± 225.5	<0.001	0.009	<0.001
ART max	187.43 ± 72.1	355 ± 51	392 ± 47	<0.001	<0.001	<0.001
D index	6.7 ± 2.5	1.4 ± 0.5	1.29 ± 0.6	<0.001	0.02	<0.001

*ART* Ambrosio relational thickness, *avg.* average; *BDE* back difference elevation, *CCT* central corneal thickness, *cont* controls *CTmin* minimum corneal thickness, *D index* multimetric D *FFKC* forme fruste keratoconus, *KCN* keratoconus *Kf* flat keratometry, *Km* mean keratometry, *Ks* steep keratometry, *max* maximum, *min* minimum, *PE* posterior elevation, *PPI* pachymetric progression index

As shown in Table 1, the keratometric reading (i.e., steep K, flat k, mean K), difference between the central corneal thickness and thinnest location, difference between the posterior corneal elevation (PE) and back difference elevation (BDE), and D index measurement were significantly higher in eyes with keratoconus than in eyes of normal control subjects; the corneal thickness and Ambrósio relational thickness (ART avg., ART min, ART max) were significantly lower in eyes with keratoconus than in eyes of normal control subjects (*P* < 0.05).

There was no statistically significant difference in the steep K, flat K, difference between pachymetry at the thinnest point and the apex, PPIavg, and PPI min between the forme fruste eyes (fellow eyes) and the control group. The mean K, corneal pachymetry, BDE, PE, PPImax, Ambrósio relational thickness (ARTmax, ARTmin), and multimetric D were statistically significant in the forme fruste eyes compared to the control group.

The receiver operating characteristic curve analysis included the AUC, standard error,95%CI,significance level, best cutoff points, sensitivity and specificity for each parameter to differentiate between the forme fruste and control groups; these data are shown in Table 2.

**Table 2:** Roc curve analysis for the ability of different parameters to differentiate between forme fruste keratoconus eye and control eyes

parameter	AUC	SE	95%CI	*P* value	cutoff	sensitivity	specificity
Ks	0.6	0.092	0.42,0.78	0.353	42.5	64%	50%
Kf	0.5	0.091	0.3,0.6	0.93	NA	NA	NA
Km	0.82	0.06	0.68,0.95	0.353	42.4	64%	80%
CCT	0.58	0.1	0.38,0.77	0.446	NA	NA	NA
CT min	0.61	0.6	0.4,0.8	0.3	516	60%	80%
PE	0.45	0.129	0.02,0.07	0.67	NA	NA	NA
BDE	0.60	0.094	0.41,0.77	0.38	3.5	53%	80%
PPI avg	0.404	0.016	o.19,o.61	0.37	NA	NA	NA
PPI max	0.64	0.093	0.64,0.83	0.174	1.31	50%	70%
ART avg	0.57	0.10	0.36,0.78	0.486	NA	NA	NA
ART min	0.56	0.10	0.35,0.77	0.551	NA	NA	NA
ART max	0.88	0.056	0.77,0.99	< 0.001	404	82%	70%
D index	0.67	0.102	0.47,0.87	0.097	1.2	71%	60%

*ART* Ambrosio relational thickness, *AUC* area under curve, *avg.* average, *BDE* back difference elevation, *CCT* central corneal thickness, *CI* confidence interval, *CT min* minimum corneal thickness, *D index* multimetric D, *Kf* flat keratometry, *Km* mean keratometry, *Ks* steep keratometry, *max* maximum, *min* minimum, *NA* not applicable,*SE* standard error, *PE* posterior elevation, *PPI* pachymetric progression index

**Fig. 1:**
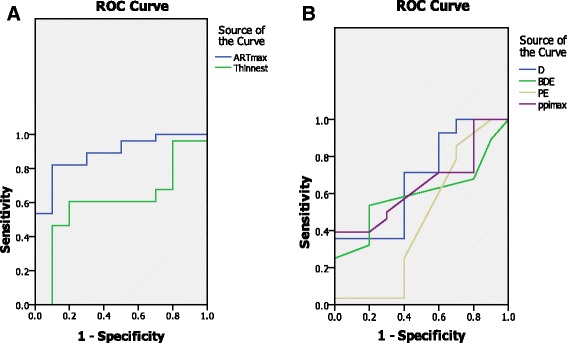
**a** ROC curve for Ambrósio relational thickness (ART max) and thinnest pachymetry**. b** combined ROC curves of multimetric D index(D), back difference elevation (BDE), Posterior elevation(PE) and pachymetric progression index maximum (PPI max) between the forme fruste and control cases

As shown in Table 2, there was a large AUC for the mean K, thinnest pachymetry, ART max, BDE, D index, and PPI max. However, the AUC was the greatest for the ART max (0.88), Fig. 1.

**Fig. 2:**
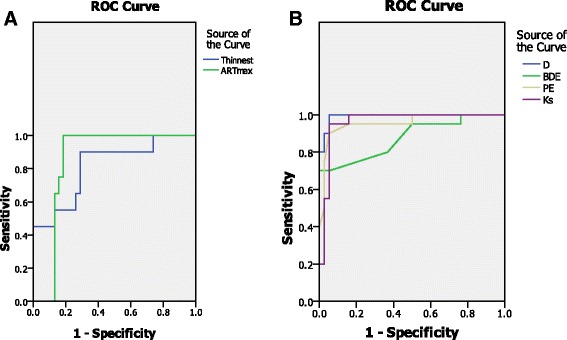
**a** ROC curve for Ambrósio relational thickness (ART max) and thinnest pachymetry. **b** combined ROC curves of multi metric D index(D), back difference elevation (BDE), Posterior elevation (PE) and steepest Keratometry (Ks) between the clinical keratoconus and control cases

The ROC curve analysis of the Scheimpflug parameters to differentiate the clinical keratoconus group from the control group was shown in Table 3 and Fig. 2.

**Table 3:** ROC curve analysis for the ability of Scheimpflug parameters to differentiate keratoconus eye from control

parameter	AUC	SE	95% CI	*P* value	cutoff	Sensitivity	specificity
Ks	0.96	0.025	0,1	< 0.001	43.6	95%	90%
Kf	0.65	0.3	0.4,0.8	0.054	41.7	70%	55%
Km	0.94	0.089	0.8,1	< 0.001	42.9	75%	90%
CCT	0.65	0.081	0.052	< 0.001	524	65%	71%
CT min	0.81	0.060	0.96,0.93	< 0.001	516	90%	82%
PE	0.95	0.029	0,1	< 0.001	9.5	90%	95%
BDE	0.87	0.053	0.77,0.97	< 0.001	12.5	70%	98%
PPI avg	0.93	0.034	0.8,1	< 0.001	1.2	80%	95%
PPI max	0.98	0.011	0,1	< 0.001	1.5	100%	95%
ART avg	0.94	0.036	0.83,1	< 0.001	368	80%	98%
ART min	0.88	0.051	0.78,0.98	< 0.001	568	75%	80%
ART max	0.85	0.055	0.74,0.96	< 0.001	330	100%	82%
D index	0.99	0.007	0,1	< 0.001	2.07	100%	95%

*ART* Ambrosio relational thickness, *AUC* area under curve, *avg.* average, *BDE* back difference elevation, *CCT* central corneal thickness, *CI* confidence interval, *CTmin* minimum corneal thickness, *D index* multimetric D, *Kf* flat keratometry, *Km* mean keratometry, *Ks* steep keratometry, *max* maximum, *min* minimum, *NA* not applicable,*SE* standard error,*PE* posterior elevation, *PPI* pachymetric progression index

In discriminating between the keratoconus eyes and control group, almost all parameters had a large AUC. The D index had the greatest AUC, followed by posterior elevation, BDE, and ART max.

The visual acuity, spherical equivalent and outcome of the contrast and sensitivity test are illustrated in Table 4.For the contrast sensitivity test with glare, there were statistically significant differences between the forme fruste eyes and the control group (*P* < 0.01); for the contrast sensitivity test without glare, there were no significant differences between the forme fruste eyes and the control group (*P* < 0.06).For both contrast sensitivity tests, there were significant differences between the clinical keratoconus eyes and the control group.

**Table 4:** Visual acuity, refraction and contrast sensitivity outcome

test	KCN	FFCK	Control	KCN vs control	FFKC VS control	KCN vs FFKC
CDVA	0.8 ± 0.2	1.03 ± 0.15	1.13 ± 0.1	<0.001	0.06	0.05
SE	−5.05 ± 4.5	−1.8 ± 2.8	−1.5 ± 2.1	<0.001	0.07	<0.01
Contrast sensitivity (%)	45.6% ± 8.2	65.6 ± 4.5	81.3 ± 5.2	<0.001	0.06	0.04
Glare(%)	41.5% ± 6.6	62 ± 5.2	77.2 ± 6.4	<0.001	<0.001	0.2

*CDVA* corrected distant visual acuity, *KCN* keratoconus, *FFKC* forme fruste keratoconus, *SE* Spherical equivalent

## Discussion

It has been established that keratoconus is a bilateral progressive disease with strong genetic association [7].It is difficult to make a diagnosis of early keratoconus, as there is no clear definition of these entities in the literature. Many terms have been employed to describe the preclinical stages of the KC condition, including subclinical keratoconus, keratoconus suspect and forme fruste keratoconus [9, 10].We based our study on the Saad and Gatinel [10]definition of forme fruste keratoconus, which defines it as the contralateral eye of unilateral keratoconus without any ectatic changes clinically or topographically using Placido disk-based topography; the definition has been followed by many researchers [10–13].

The introduction of the rotating Scheimpflug camera has introduced many diagnostic parameters, such as keratometric, pachymetric or elevation parameters. In this study, ROC curves were analyzed to detect the specificity and sensitivity of each parameter; moreover, the quality of mesopic vision was subjectively assessed to identify the sensitive and specific detectors, which support early and accurate diagnoses of forme fruste cases. The ART max had the largest area under the curve in forme fruste eyes compared to the control group, followed by the multimetric D index. This result does not agree with that of Muftuoglu et al., [14] who reported that the multimetric D had the largest AUC in cases of forme fruste keratoconus (used as simulation group of the subclinical cases).The discrepancy may be explained by different statistical analyses as the D index became more diagnostic when increased; however, the ARTmax became more diagnostic when decreased. We found an ARTmax cutoff value of 404 with a sensitivity and specificity of 82 and 70%, respectively. However, the D index cutoff was 1.2 with a sensitivity and specificity of 71and 60%, respectively, in cases of forme fruste keratoconus. In other words, the false negative results are rare when applying ART max; however, there is limited sensitivity and specificity when applying the multimetric D index, with possible false-positive and false-negative results in forme fruste cases. In clinical cases, the multimetric D had a larger AUC compared to the ART max; this result may be due to the multimetric D index being a multimetric parameter composed of keratometric, pachymetric, pachymetric progression and back elevation parameters [14], which change significantly in clinical keratoconus cases. For the forme fruste cases in this study, the largest AUC was found for the mean K, followed by PPI and back difference elevation, while the posterior elevation had a low AUC. This is similar to the findings of previous studies [6, 10], which indicated that the back difference elevation is better for diagnosing subtle cases of keratoconus in normal eyes compared to the back elevation. In our study, the sensitivity and specificity of the posterior elevation and back difference elevation were limited; this result is supported by the findings of Muftuoglu et al. [6], who stated that there was significant overlap of the back difference elevation and posterior elevation between forme fruste eyes and control eyes.

In our study, the posterior elevation was not found to be a sensitive parameter in detecting the forme fruste cases; this result disagrees with reports of Muftuoglo et al. [6], which may be due to most of the keratoconus eyes being mild to moderate cases, and the fellow eyes showed minimal changes in the early stage.

The corneal thickness and thinnest corneal points had large AUCs for diagnosing keratoconus, which agrees with previous studies [15–17]; however, both parameters had low AUCs in forme fruste eyes. This result may be explained by the thickness not being significantly decreased in early keratoconus, so they had higher levels and lower sensitivity.

In this study, the contrast sensitivity test with glare was found to be an important diagnostic test for early forme fruste cases. This finding is in accordance with that of Bilen et al., [18] who reported that contrast sensitivity(CS) was affected more than corrected distant visual acuity (CDVA) in its correlation with refractive, topographic, pachymetric, and aberrometric changes. This report concluded that CS is a more sensitive indicator of visual function in keratoconus follow-up than CDVA; however, Bilen et al. assessed CS by the Hamilton veal chart [19].

Maeda et al. [20] found a significantly greater loss in CS in a keratoconus group compared to a normal control group. They concluded that subtle visual deteriorations, which are detected by CS testing, maybe predicted objectively by corneal topographic indices.

## Conclusion

There is no single Pentacam parameter that can be used to diagnose forme fruste keratoconus with high specificity and sensitivity, and further studies should be conducted to determine more efficient parameters. The ART max seems to increase our detection rate of forme fruste keratoconus; however, the sensitivity and specificity of all Pentacam parameters are limited in diagnosing forme fruste keratoconus, including the ARTmax. Early detection of mesopic vision may be an easy outpatient subjective test to identify a forme fruste case and differentiate it from the true unilateral keratoconus, which despite being very rare should still be considered.

## Abbreviation

ART: Ambrosio relational thickness
AUC: Area under curve
Avg: Average
BDE: Back difference elevation
CCT: Central corneal thickness
Cs: Contrast sensitivity
CTmin: Minimum corneal thickness
D index: Multimetric D
FFKC: Forme fruste keratoconus
KCN: Keratoconus
Kf: Flat keratometry
Km: Mean keratometry
Ks: Steep keratometry
PE: Posterior elevation
PPI: Pachymetric progression index

## References

[1] Rabinowitz YZ, Nesburn AB, Mcdonnell PJ (1993). Videakeratography of the fellow eye in unilateral keratoconus. Ophthalmology.

[2] Li X, Rabinowitz YS, Rasheed K, Yang H (2004). Longitudinal study of the normal eyes in unilateral keratoconus patients. Ophthalmology.

[3] Rabinowitz YS (1998). Keratoconus. Surv Ophthalmol.

[4] Seiler T, Quurke AW (1998). Iatrogenic keratoconus after LASIK in a case of forme fruste keratoconus. J Cataract Refract Surg.

[5] Randeleman JB, Woodward M, Lynn MJ, Stulting RD (2009). Risk assessment for ectesia after corneal refractive surgery. Ophthalmology.

[6] Muftuoglu O, Ayer O, Ozulken K, Ozyol E, Akinci A (2013). Posterior corneal elevation and back difference corneal elevation in diagnosing forme fruste keratoconus in the fellow eyes of unilateral keratoconus patients. J Cataract Refract Surg.

[7] Ys R (1998). Keratoconus. Surv Ophthalmol.

[8] Rabinowitz YS, Mcdonnell PJ (1989). Computer assisted corneal topography in keratoconus. Refract Corneal Surg.

[9] Ambrósio RJ, ALC C, Guerra FP, Louzada R, Sinha Roy A, Luz A, Wj D, Belin MW (2011). Novel pachymetric parametrere based on corneal tomography for diagnosing keratoconus. J Refract Surg.

[10] SaadA GD (2010). Topographic and tomographic properties of formefrustekeratoconus corneas. Invest Ophthalmol Vis Sci.

[11] Edwards M, CNJ M, Dean S (2001). The genetics of keratoconus. Clin Exp Ophthalmol.

[12] Amsler M (1961). The “forme fruste” of keratoconus (in German). Wein Klin Wochenschr.

[13] Klyce SD (2000). Chasing the suspect: keratoconus detection with KISA% method-another view. J Cataract Refract Surg.

[14] Muftuoglu O, Ayar O, Humeric V, Orucoglu F, Kilic I (2015). Comparison of multimetric D index with keratomtric, pachymtric, and posterior elevation parameters in diagnosing subclinical keratoconus in the fellow eyes of asymmetric keratoconus patients. J Cataract Refract Surg.

[15] Gatinel D, Saad A (2012). The challenges of the detection of subclinical keratoconus at its earliest stage. Int j keratocoectatic Corneal Dis.

[16] Ambrósio RJR, Alonso RS, Luz A, LG CV (2006). Corneal thickness spatial profile and corneal volume distribution: Tomographic indices to detect keratoconus. J Cataract Refract Surg.

[17] Ucakhan OD, Cetinkor V, Ozkan M, Kanpolat A (2011). Evaluation of the Scheimpflug imging parameters in subclinical keratoconus, keratoconus, and normal eyes. J Cataract Refract Surg.

[18] BilenNB HIF, Arce CG (2016). Correlation between visual function and refractive, topographic, pachymetric and aberrometric data in eyes with keratoconus. Int J Ophthalmol.

[19] Pelli DG, Robson JG, Wilkins AJ (1988). The design of a new letter chart for measuring contrast sensitivity. Clin Vision Sci.

[20] Maeda N, Sato S, Watanabe H, Inoue Y, Fujikado T, Shimomura Y, Tano Y (2000). Prediction of letter contrast sensitivity using videokeratotopographic indices. Am J Ophthalmol.

